# Research on the Effects of the Water–Binder Ratio and Fiber Content on the Tensile and Bending Mechanical Properties of ECCs

**DOI:** 10.3390/ma18030509

**Published:** 2025-01-23

**Authors:** Jifeng Ai, Kaixin Qiu, Bowei Yang, Shiwei Peng, Qiang Zhang, Jiuhong Jiang

**Affiliations:** 1 Innovation Demonstration Base of Ecological Environment Geotechnical and Ecological Restoration of Rivers and Lakes, College of Civil Engineering, Architecture and Environment, Hubei University of Technology, Wuhan 430068, China; 102210915@hbut.edu.cn (J.A.); 102110917@hbut.edu.cn (K.Q.); 102110969@hbut.edu.cn (B.Y.); 102210925@hbut.edu.cn (S.P.); 2 China Construction Third Engineering Bureau Group Co., Ltd., Wuhan 430064, China; zhangqiang2@hbgd7.wecom.work

**Keywords:** engineered cementitious composites, water–binder ratio, fiber content, tensile toughness, energy factor *E_ms_*

## Abstract

Engineered cementitious composites (ECCs) are a type of high-performance composite material, but in practical applications, ECCs that combine high strength with high toughness have greater development potential. Moreover, there is currently no unified standard or method for assessing the bending toughness of ECCs. This study is based on the Modified Andreasen and Andersen model (MAA) design ratio of the closest packing theory to investigate the effect of changes in the water–binder ratio and fiber content, where the water–binder ratio is taken as 0.19, 0.22, and 0.25, and the fiber content is taken as 1.0%, 1.5%, and 2.0%, respectively, to improve the comprehensive performance of ECCs. Information about tensile strength, bending strength, tensile toughness, and bending toughness is examined. Three different ways to measure bending toughness are compared and improved; the energy factor *E_ms_* is used to measure bending toughness, and a response surface methodology is used to design and test the best mix ratio. The results indicate that when the water–binder ratio is 0.22 and the fiber content is 1.9%, the performance is optimal. Compared with the prediction group, the measured group presented an increase in tensile strength of 2.64%, a decrease in bending strength of 3.39%, an increase in tensile toughness of 3.22%, and an increase in the energy factor *E_ms_* of 1.21%. This finding indicates that the response surface optimization improved the performance of the ECCs in various aspects.

## 1. Introduction

Engineered cementitious composites (ECCs) are high-ductility, fiber-reinforced cementitious composites pioneered and designed by Li V C. of the University of Michigan, USA [1,2,3,4]. A multitude of studies have demonstrated that Polyethylene Fiber (PE) ECCs have the ability to stabilize and control the development of cracks, which is reflected mainly in the obvious strain hardening characteristics of the sample after the incorporation of PE fiber and the ultimate tensile strain of the maximum range of 3% to 12%, which solves, to some extent, the brittleness, inferior toughness, poor durability, and other problems of cementitious materials [5,6,7,8].

Wang [9] et al. investigated the effect of PE fibers at different fiber contents (0%, 1%, 1.5%, and 2%) on the fine and macrobehavior of ECCs. The slip-hardening behavior of the fiber–matrix interface of PE fibers during the tensile process was demonstrated via single-fiber pullout tests, and axial tensile tests revealed that the ECC still had good strain-hardening properties at a fiber content of 1% and that the fiber bridging properties increased and then decreased with increasing fiber content. Yu [10] and others prepared a highly ductile ECC using PE fibers as the main external reinforcing fibers, achieving an average tensile strain of 8%, with some maximum tensile strains exceeding 12%. The crack spacing is less than 2 mm, the average crack width is less than 100 μm, and the compressive strength reaches 45.9 MPa to 121.5 MPa, allowing the ECC to maintain durability even at extreme values. Mohamed Maalej [11] et al. prepared ECCs using a blend of high-modulus steel fibers and relatively low-modulus PE fibers. Owing to the relatively small fiber aspect ratio, the ECCs produced were not as durable as the PE fibers, resulting in the preparation of ECCs with high tensile strength but low tensile strain. Although the blending of steel fibers can improve the tensile strength of ECCs, their tensile strain is not consistent with the high strain and strain hardening characteristics of ECCs. Li V C. [12] et al. reported that when a certain amount of PE fibers were blended into ECCs, the compressive strength of the samples decreased to different degrees, possibly because the addition of fibers disrupted the internal pore environment of the matrix, and simultaneously, the increased incorporation of fibers led to a greater number of internal pores, resulting in a decrease in the density of the matrix, which caused a reduction in the compressive strength. Cheng [13] et al. investigated the effects of changing the water–binder ratio on various properties of FRCCs at 7 d and 28 d. They reported that when the water–binder ratio was lower than 0.22, the rheological properties of the samples deteriorated, the internal pore space of the samples decreased, and the pore diameter and volume increased, which further reduced the mechanical properties of the samples.

Bending performance, as one important evaluation indicator for cement-based materials, has attracted the attention of numerous researchers in recent years, particularly regarding the effects of fibers on the bending toughness of fiber-reinforced concrete [14,15,16]. On this basis, many researchers have begun to investigate the bending performance and bending toughness of ECCs, which is also important for their practical engineering applications and development [8,17,18]. Li [19] and others used polypropylene fibers and steel fibers, respectively, to analyze the effect on the bending toughness of lightweight aggregate fiber-reinforced cementitious materials and used the ASTM C1609 [20] and JSCE SF-4 [21] toughness evaluation methods to assess their bending toughness. The test results revealed that the addition of 1.1% polypropylene fibers and 2% steel fibers enhanced the bending toughness properties better than the addition of polypropylene fibers did. The postpeak ductility behavior of the polypropylene fibers was better than their prepeak behavior, but the prepeak ductility behavior of the steel fibers was better than their postpeak behavior. Suthiwarapirak [22] and colleagues conducted a study of flexural fatigue tests of PE-ECCs and PVA-ECCs. Their findings revealed that the ultimate bending strength of samples measuring 100 mm by 100 mm by 400 mm was 9.93 MPa, whereas the ultimate flexural deflection was 8.71 MPa. Similarly, the ultimate bending strengths of the samples measured 100 mm by 100 mm by 400 mm were also 9.93 MPa and 8.71 MPa, respectively. Some of the largest bending deflections were 2.5 mm and 1.5 mm, respectively. In the bending fatigue test, there were fewer broken fibers and pulled-out fibers in the PE-ECC than in the PVA-ECC. This finding shows that the PE-ECC has better bending strength and deflection capacity. Hamid Reza Pakravan [23] investigated the bending and compressive resistance of PE-ECCs by blending two types of fibers with high and low moduli. The results revealed that hybridization of fibers with high chemical bonding to the cement matrix adversely affects the mechanical properties of cement concrete, and significantly reduces the mixture ease and ductility. PE fibers reduce the bending strength of hybrid composites because of their lack of chemical adhesion and lower modulus of elasticity. However, hybridization of PVA fibers with a lower modulus of elasticity produces a synergistic effect such that the hybrid strength of the ECC decreases slightly, but the plasticity further increases to 148%, and fiber hybridization can effectively improve the ductility of the ECCs. Kâzim Turk [24] evaluated the 3 d, 28 d, and 90 d bending toughness of ECCs by using ASTM C1609, JSCE-SF4, and the postcracking strength method to investigate the effect of cement replacement using fly ash and limestone filler on ECCs. The experimental results show that the flexural load-bearing capacity and ductility of the 3d-ECC are optimal when the ratio of fly ash to cement is 3.2. Zhang [25] proposed an improved method for evaluating the bending toughness of ECC materials because the JSCE-SF4 method, the ASTM C1609 method, and the postcracking strength (PCS) method cannot be used for the comprehensive evaluation of ECCs with high toughness and high flexural capacity. The method proposes two new indices: equivalent initial flexural and postpeak bending toughness indices and a new index as an initial energy factor. This new evaluation method adapts to the large deflection deformation of the ECCs, eliminates the determination of the first crack point, and is more commonly used for evaluating the bending toughness of the ECCs. Long [26] et al. investigated the flexural performance and fatigue performance of a PE-ECCs by analyzing its bending performance under an ultralow-temperature environment and a freeze–thaw cycle. The results show that these conditions greatly affect the internal porosity of the PE-ECC matrix, reducing the ability of the sample to undergo multiple-seam cracking. Research on the durability performance of PE-ECCs involves more comprehensive analysis and testing, providing a certain theoretical basis for practical applications. Yang [27] et al. used millimeter-scale polyethylene (PE) fibers, micron-scale calcium carbonate whiskers (CWs), and nanometer-sized carbon nanotubes (CNTs) as externally doped fibers to form a multiscale reinforcement system, and they tested the effects of multiscale fibers on the mechanical properties of ECCs. The results indicated that doping the ECC matrix with 1.55% PE fibers, 2.17% CWs, and 0.154% CNTs optimized the compressive strength, bending strength, and tensile strength, respectively.

Based on the analysis of the above research results, single variable, external dopant or fiber blending methods are used to improve the mechanical properties of ECCs. However, many factors affect the mechanical properties of ECCs, especially the water–binder ratio and fiber blending. With respect to Zhang’s [25] proposed method for improving the blending toughness evaluation of ECCs materials, there are also several limitations. Thus, a more comprehensive toughness analysis method is needed. In modern buildings and infrastructures, which have high requirements for the whole life cycle of the structures and the performance and durability of the structural foundation, ECCs with both high strength and high toughness will have a wide range of applications. However, relatively few studies have evaluated the high strength and toughness of ECCs.

In this work, using the closest packing theory to design the test ratio, the effects on the tensile and bending properties of ECCs are investigated by taking the water–binder ratio and fiber content as variables, and the test groups with optimal tensile strength, tensile toughness, bending strength and its toughness, respectively, are obtained. By comparing and analyzing the advantages and disadvantages of the three bending toughness evaluation methods, an improved toughness evaluation method is proposed. Finally, through the use of Design-Expert 12 software, we establish a regression model to demonstrate the tensile strength, tensile toughness, bending strength, and bending toughness as the response value. We then analyze the interaction of the two factors. Through the response surface method of multi-objective optimization of the design of the mix ratio, we derive the predicted test group with optimal comprehensive performance. The final purpose of combining high strength and high toughness of ECCs was realized.

## 2. Materials and Methods

### 2.1. Materials

The raw materials used in the preparation of ECCs include PO42.5 ordinary silicate cement, research grade fly ash (FA), test grade silica fume (SF) and quartz sand featuring a uniform gradation. All of these materials are of Chinese origin. Since the closest packing theory is a packing model with a continuous distribution of particle size, a particle size analyzer was used in the test to analyze the particle size distribution of the above four materials, resulting in a range of particle sizes as shown in Table 1. Table 2 shows the performance indicators of PE fibers with a high modulus of elasticity. The water-reducing agent is a polycarboxylic superplasticizer, which has a 25% water reduction rate. The water used in this test was ordinary tap water.

### 2.2. Mix Designs

The Modified Andreasen and Andersen model (MAA) of the most compact accumulation theory [28] was used to design the mixing ratio for this test via the following design equations:

(1) $$P\left(D_{i}\right)=\frac{D_{i}^{q}-D_{min}^{q}}{D_{max}^{q}-D_{min}^{q}}$$

In the formula: *P(D_i_)* is the cumulative particle size fraction (%) less than *D_i_*

*D_i_* is the particle size(mm),

*D_max_* is the maximum particle size(mm),

*D_min_* is the minimum particle diameter(mm),

*q* is the distribution coefficient.

**Figure 1:**
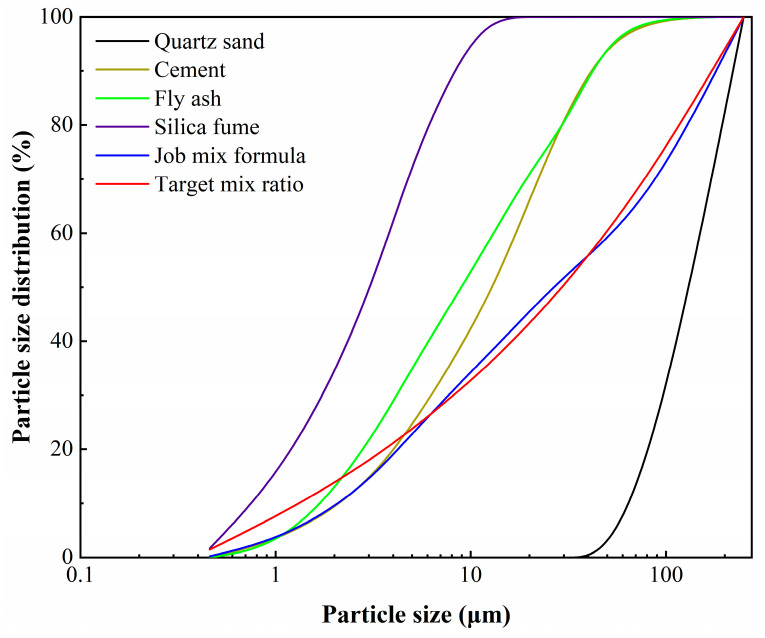
Particle size distribution.

Plotting the particle size distributions of various materials and solving the target curve (e.g., red curve in Figure 1) allows us to adjust the allocation ratio of each material via the least squares method. The closeness of the fit ratio to the target curve was assessed by the residual sum of squares formula. The formula for calculating the residual sum of squares (RSS) is as follows:

(2) $$RSS=\overset{n}{\underset{i=1}{\sum }}{\left(P_{mix}\left(D_{i}^{i+1}\right)-P_{tar}\left(D_{i}^{i+1}\right)\right)}^{2}$$

where *P_mix_* is the mixing ratio of the materials.

*P_tar_* is model calculation of target mix ratio

For this test, the chosen mixture ratio was volume ratio of cement: fly ash: silica fume: quartz sand, which was 0.43:0.18:0.03:0.36, the performance indices of these four materials, respectively, as shown in Table 3, Table 4, Table 5 and Table 6. A water reducing agent content of 0.1% of the mass of cementitious materials. Water–binder ratios of 0.19, 0.22, and 0.25, and fiber contents of 1%, 1.5%, and 2% were selected as variables to design nine groups of tests, and test fits were shown in Table 7.

### 2.3. Test Methods

#### 2.3.1. Tensile Test Method

**Figure 2:**
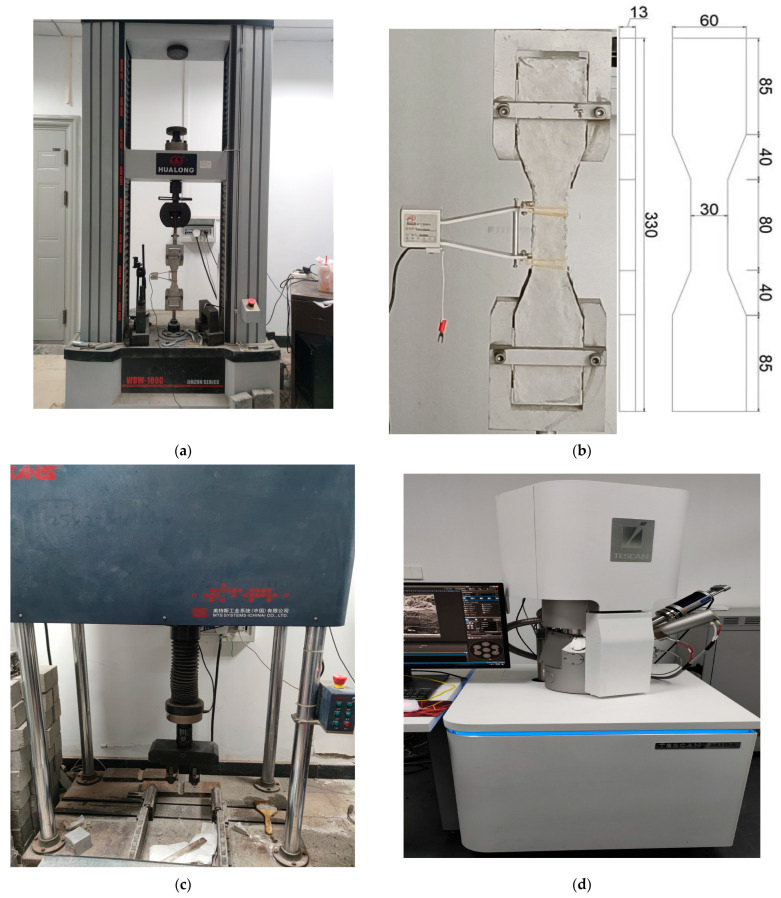
Test Apparatus. (**a**) WDW-100CDE type microcomputer control electronic universal testing machine. (**b**) Tensile test sample. (**c**) MTS Microcomputer Control Electronic Pressure Tester. (**d**) scanning electron microscope (SEM).

The tensile test was conducted according to JG/T2461-2018 [29]. The test was conducted using dog bone samples for tensile testing with a loading rate of 0.5 mm/min. During the tensile test, the tensile deformation of the tensioned section was recorded via an extensometer, and the stress–strain diagrams were plotted. The test apparatus and the dimensions of the dog bone are shown in Figure 2a and Figure 2b.

#### 2.3.2. Bending Test Method

The bending test was conducted according to JG/T2461-2018 [29]. Bending tests were used a prism with dimensions of 100 mm × 100 mm × 400 mm and a loading speed of 0.05 mm/min. The load and mid-span deflection were recorded during the bending test, and the load–deflection diagram was plotted. Figure 2c shows the test apparatus.

#### 2.3.3. Microscopic Test Method

Scanning electron microscopy (SEM) was used to visualize the degree of hydration of the ECC matrix, fiber dispersion, fiber/base interface layer, and other microscopic morphology. A Czech TESCAN MIRA LMS type scanning electron microscope was used, as shown in Figure 2d.

### 2.4. Sample Preparation

**Figure 3:**
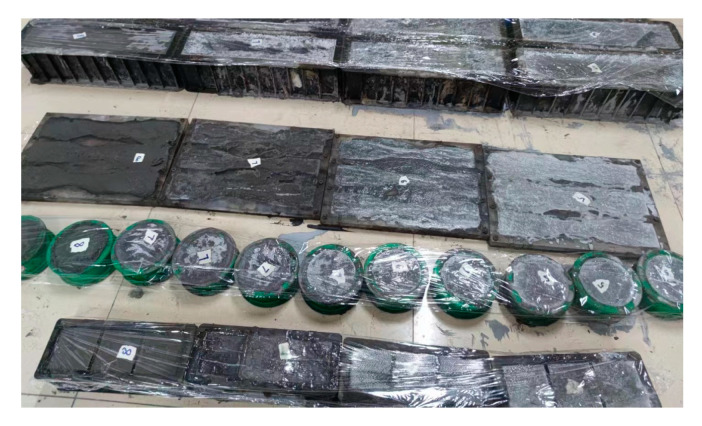
Sample preparation.

The preparation process was as follows: (1) cement, FA, and SF were poured into the mixer and dry mix for 2 min to mix fully. (2) Quartz sand was added to the dry mixture, which was mixed for 2 min. (3) Water and a polycarboxylic superplasticizer water reducing agent were evenly dispersed to form a mixture, which was poured into the mixer and mixed for 3 min to fully react. (4) PE fibers were dispersed by hand and evenly added to the mixer for 2 min. (5) The finished cement mortar was poured into the mold, placed on the vibration table, and vibrated for 5 min until it was dense. One day later, the mold was shaped, demolded, and placed in the standard curing room (temperature of 20 ± 2 °C and minimum humidity of 96%) for 28 d. Figure 3 displays the sample preparation form.

## 3. Results

### 3.1. Analysis of Tensile Test Results

#### 3.1.1. Tensile Strength

In this axial tensile experiment, the effective load in Equation (3) was used as tensile stress. Equation (4) converts the deformation index to tensile strain. The equations are shown below:

(3) $$\sigma =\frac{F}{A}$$

where σ is the stress, *F* is the effective load, and *A* is the cross-sectional area of the test section.

(4) $$\varepsilon =\frac{L_{1}-L_{0}}{L_{0}}$$

where ε is the strain, *L_1_* is the elongation of the test section after stretching, and *L_0_* is the initial length of the test section.

**Figure 4:**
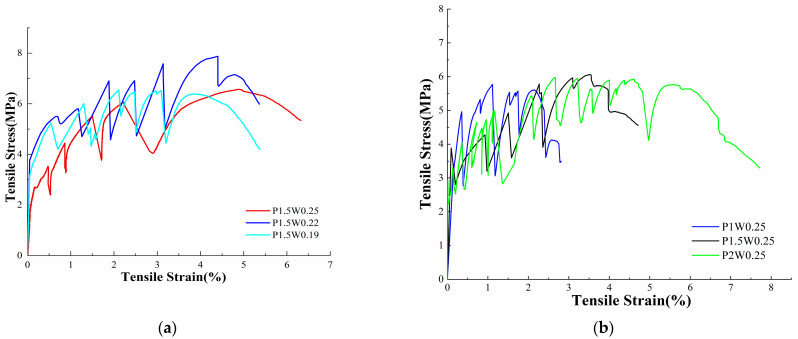
ECC axial tensile stress–strain diagrams. (**a**) Stress–strain diagrams for different water–binder ratios. (**b**) Stress–strain diagrams for different fiber content.

Figure 4 shows the stress–strain curves of the ECC samples after 28 d of curing, and the tensile samples exhibited strain-hardening characteristics, which were evaluated in terms of the magnitude of sample elongation. As shown in Figure 4a,b, when the fiber content was 1.5%, the elongations of the test groups with water–binder ratios of 0.19, 0.22, and 0.25 were 4.82%, 5.09%, and 6.13%, respectively, and the growth rate of the ultimate elongation was 27.18%, which indicated that the elongation increased with the increasing water–binder ratio. When the water–binder ratio was 0.25, the ultimate elongations of the test samples with 1.0%, 1.5%, and 2.0% fiber contents were 2.29%, 3.98%, and 6.55%, respectively, with an elongation growth rate of 186.03%. These results indicate that the fiber content has a great effect on the growth of the elongation of the ECC, whereas the water–binder ratio has a limited effect on the growth of the ECC elongation. With the increasing hydrogel ratio, the internal matrix/fiber bonding resistance of the sample decreases, the fibers are pulled and slipped, the internal bridging performance of fibers in the matrix increases, and thus, the elongation of the sample increases. With increasing fiber content, the internal structure of the sample with a high water–binder ratio is relatively loose, and the high modulus of elasticity of the fibers at the cross-section emphasizes the characteristics of the fibers, The greater the fiber content is, the stronger the ability of the sample to resist tensile deformation, so the P2W0.25 test group shows excellent strain hardening characteristics.

**Figure 5:**
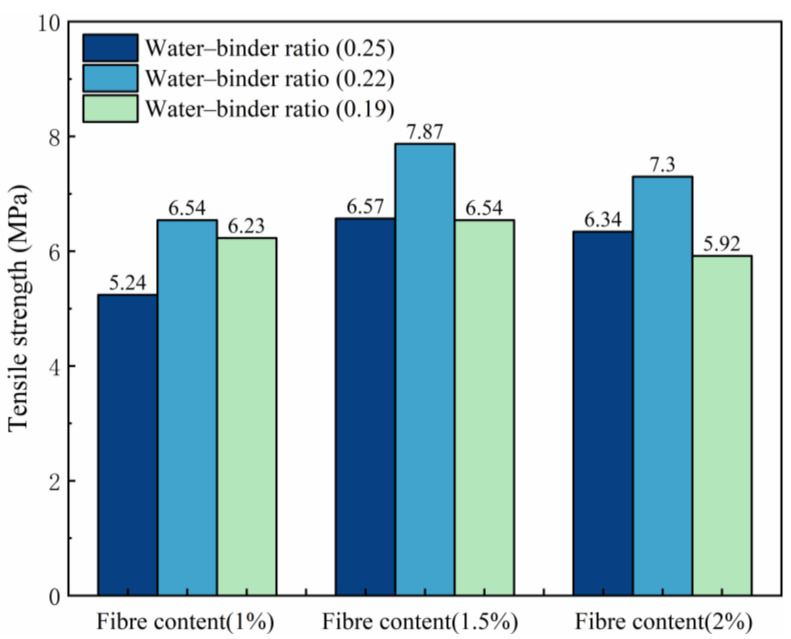
Effects on tensile strength.

The tensile strength change in trend is shown in Figure 5. With the reduction in the water–binder ratio, the tensile strength of ECC increases and then decreases. It also shows a water–binder ratio of 0.22 for reaching the maximum tensile strength and a P1.5W0.22 test group for reaching the maximum tensile strength of 7.87 MPa. Compared with the P1.5W0.19 test group, the growth rate of its tensile strength is 19.79%. This finding shows that when the water–adhesive ratio is 0.22, the sample matrix cross-section and fiber bonding force reach the optimal range, and the fibers are well dispersed to facilitate the preparation of air discharge. When the water–binder ratio is greater than 0.22, the internal compactness of the sample is reduced, the bonding resistance between the fiber and matrix is weakened, and the fiber is easily pulled out of the damaged interface, which leads to a reduction in tensile strength. When the water–binder ratio is less than 0.22, the sample is relatively dense, which is not conducive to fiber dispersion, and fiber agglomeration in the matrix easily results in the formation of bubbles. The sample develops rapidly along the internal pore space to generate cracks under tensile action, resulting in a reduction in tensile strength.

With increasing fiber content, the tensile strength of ECC increases but then decreases, with a fiber content of 1.5% reaching the maximum tensile strength. Compared with that of test group P1.5W0.22, the tensile strength of the test group P1.0W0.22 increases, with a tensile strength increase rate of 20.34%. This is because the fibers are uniformly distributed inside the matrix, and the high elastic modulus characteristics of the fibers are fully utilized. When the fiber content is less than 1.5%, the distribution of fibers in the damaged cross-section is less, and the ability to inhibit the development of cracks is limited. When the fiber content is greater than 1.5%, the fiber overdose leads to the tensile region of the fiber agglomerates and uneven distribution of the sample, resulting in a concentration of stress at the interface. Simultaneously, the fiber chaotic distribution leads to the cross-section of the bubble being difficult to discharge, resulting in harmful porosity, which leads to the tensile strength of the sample being significantly reduced.

#### 3.1.2. Tensile Toughness

The water–binder ratio and fiber content will affect the tensile strength to different degrees. While the tensile toughness is important for evaluating the tensile properties of ECCs, this section evaluates the tensile toughness of ECCs by integrating the stress–strain diagrams with the *X*-axis enclosing the area and introducing the strain energy g as the ability of the sample to absorb energy [30]. The calculation formula is as follows:

(5) $$g=\int_{0}^{\varepsilon u}\sigma \left(\varepsilon \right)d\left(\varepsilon \right)$$

where g represents the strain energy, which is the energy absorbed by the matrix before the destruction of the sample; σ represents the tensile stress; ε represents the tensile strain; and ε_u_ represents the tensile strain corresponding to the reduction in the tensile strength to 85% of the ultimate tensile strength. The peak value is the extreme point in the stress–stress curve, which is the point of maximum stress. The peak elongation is the peak strain value in the stress–stress curve. The postpeak elongation is the difference between the peak strain and the strain corresponding to the last descending curve from the peak strain to 0.85 times the peak stress. When the stress value reaches 0.85 times the peak stress or reaches the postpeak elongation, it can be regarded as failure and the experiment can be stopped.

**Figure 6:**
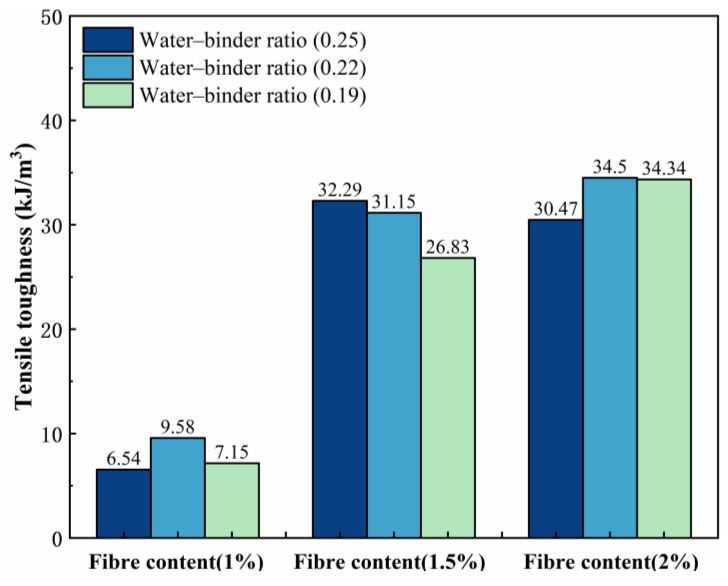
Effects on tensile toughness.

Figure 6 shows the variation pattern of the tensile toughness. The fiber content has a significant effect on the growth of the tensile toughness of ECCs. With increasing fiber content, the tensile toughness of the sample as a whole tended to increase and reached a maximum value of 34.5 kJ/m^3^ in P2W0.22. Compared with that of the P1W0.22 test group (9.58 kJ/m^3^), the growth rate of its tensile toughness was 260.12%. However, compared with that of the P1.5W0.22 test group (31.15 kJ/m^3^), the growth rate was only 10.75%. This is because a moderate amount of fibers can fully utilize the high modulus of elasticity of PE fibers to increase the ability of ECCs to absorb energy and resist deformation. However, excessive fiber incorporation leads to severe fiber agglomeration and the formation of harmful pores, which severely restricts the formation and development of the tensile toughness of ECCs.

**Figure 7:**
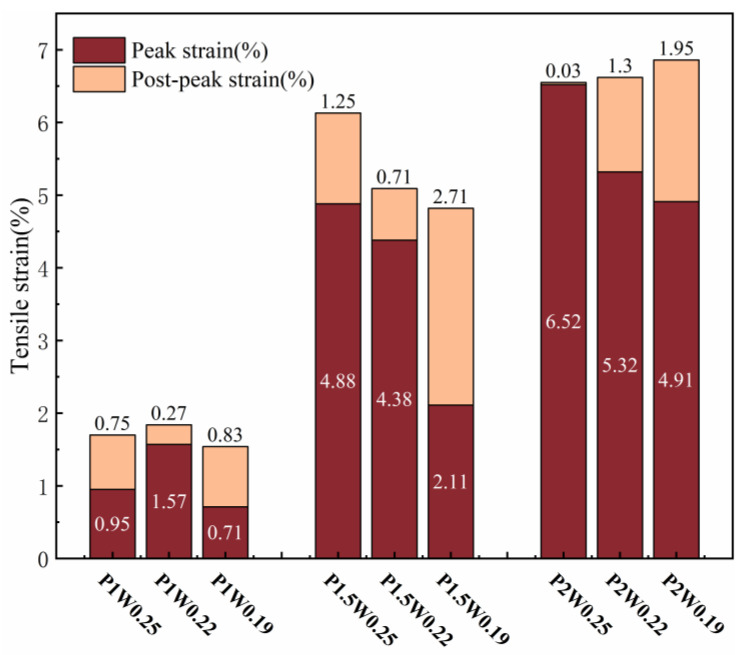
Elongation variation graph.

Figure 7 shows the variation law of elongation. When the fiber content remains constant, the trends of tensile toughness and ultimate elongation exhibit approximate similarity. Evidently, fiber content exerts a remarkable influence on the growth of elongation in ECCs.

When the fiber content is 1%, the three water–binder ratios do not have tensile toughness values greater than 10 kJ/m^3^ and 1% elongation, because the fiber content is not sufficient, and the matrix is relatively dense, the fiber–matrix cross-section bonding is too high, and most of the fibers subjected to pull-off damage cannot play a full role in the characteristics of the high modulus of elasticity of the PE fibers.

Compared with a fiber content of 1.5%, a fiber content of 1% has greater tensile toughness and ultimate elongation growth, resulting in better tensile toughness and elongation. This is because the appropriate amount of fiber content allows for bridging at the crack cross-section, most of the fibers are subjected to pull-out damage, and the high modulus of elasticity of PE fibers results in the characteristics of a high modulus of elasticity. At a water–binder ratio of 0.25, when the tensile toughness and elongation performance are better, the combination of fibers and the matrix are in the optimal state.

Figure 6 and Figure 7 show a small change in tensile toughness and ultimate elongation at 2% fiber content, with no significant increase compared with those at 1.5% fiber content. Although the reduction in the water–binder ratio improved the fiber–matrix bond, the increase in fibers led to the formation of pores in the matrix that were not easily dispersed, which reduced the tensile strength, and the high modulus of elasticity of the fibers improved the ability of the sample to absorb the energy. Thus, its tensile toughness and elongation were slightly greater than those of the sample with a fiber content of 1.5%.

#### 3.1.3. Microscopic Test

**Figure 8:**
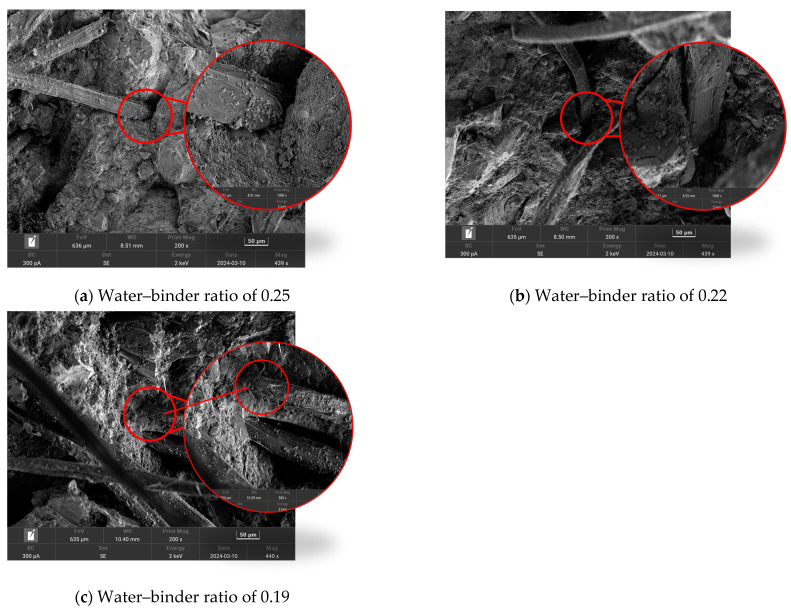
Scanning electron micrographs of tensile damage.

In order to investigate the fiber destruction morphology during the ECC tensile process, the fiber destruction morphology under a different water–binder ratio was observed by scanning electron microscopy (SEM). As shown in Figure 8a, when the water–binder ratio is 0.25, the pores generated by bubbles next to the fiber destruction are obvious, indicating that the matrix has not reached a denser state. At this time, the fibers attached to the surface of the gelling material significantly less and produce small scratches on the surface of the fibers, which indicates that the reduction in the water–binder ratio makes the matrix become dense, and the fiber/matrix cross-section bonding force increases, which improves the tensile strength.

As shown in Figure 8b, when the water–binder ratio is 0.22, the pores produced by the air bubbles next to the fibers are obviously smaller at this time, and the matrix is observed to be denser in the rest of the range. As shown, the destruction of the cross-section of the fiber half connected to the matrix, the other half of the surface damage is serious, which indicates that when the water–binder ratio is reduced to 0.22. When the destruction of the cross-section of the fiber was half pulled-out, half pulled-off, it showed a good use of the fiber’s high modulus of elasticity, thus, maximizing the enhancement of tensile strength.

As shown in Figure 8c, when the water–binder ratio is 0.19, we can observe the shooting cross-section without obvious bubbles, compared with the water–binder ratio of 0.22, which results in a denser matrix. The destruction of the cross-section of the fiber pull-off damage is obvious, and most of the fibers show pull-off damage. This is because the water–binder ratio is too low, and the fiber–matrix cross-section is too tightly bonded, therefore, the fiber cannot be pulled out of the matrix during the destruction process, resulting in fiber pull-off damage, and it cannot fulfill the characteristics of the high elastic modulus of the PE fiber. The scanning electron microscopy results show that when the water–binder ratio decreases, the destruction of the fiber pattern is as follows: most of the pull-out; half of the pull-out; half of the pull-off; a little pull-out; most of the pull-off. This adheres to the development of the law, which is consistent with the rule of change in the tensile strength. This phenomenon also shows that in a certain range, reducing the water–binder ratio will have an enhanced effect on the tensile strength, but the low water–binder ratio will have a side effect on the tensile strength.

### 3.2. Analysis of the Bending Test Results

#### 3.2.1. Bending Strength

The following formula calculates the bending strength in accordance with the bending test process and test specification requirements for testing:

(6) $$f_{1}=\frac{FL}{bh^{2}}$$

where *f_1_* is the bending strength value of the bending sample, *F* is the damage load value of the bending sample, and *L* is the clear span between the supports.

**Figure 9:**
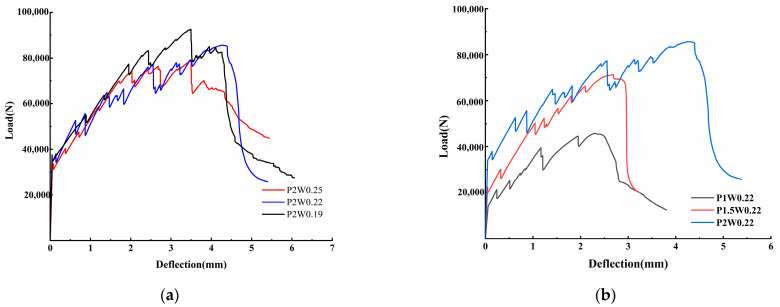
Load–deflection diagram for the ECC bending test. (**a**) Load–deflection diagrams for different water–binder ratios. (**b**) Load–deflection diagram with different fiber content.

The bending test load–deflection curves are shown in Figure 9, and the curve change process can be approximately divided into three phases: (1) elastic phase; (2) bend hardening phase; and (3) postpeak damage phase. When the fiber contents are both 2%, the change in the water–binder ratio does not obviously affect the increase in the peak load or deflection of the ECC in Figure 9a. However, when the water–binder ratio was 0.22, the peak load and deflection of the ECC significantly increased with increasing fiber content in Figure 9b.

**Figure 10:**
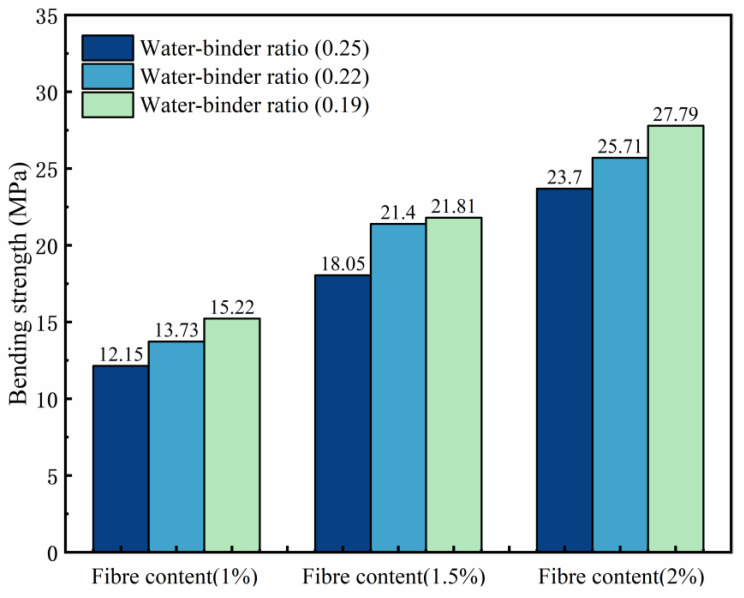
Effects on bending strength.

The influence law of the ECCs bending strength is shown in Figure 10. The bending strength gradually increases with the decreasing water–binder ratio, and the P2W0.19 test group reaches the maximum bending strength of 27.79 MPa; compared with 23.7 MPa in the P2W0.25 test group, the growth rate is 17.26%. The bending strength of the samples with the same water–binder ratio increases with increasing fiber content and reaches the maximum value at P2W0.19; the growth rate is 82.59% greater than that of the P1W0.19 test group. This finding indicates that an increase in fiber content has a significant effect on the increase in the ECCs bending strength. However, when the water–binder ratio is 0.19, the fluidity of the matrix becomes poor, the fibers are unevenly dispersed and entangled with each other internally during preparation, concentrated stress is formed during destruction, and the growth rate of the bending strength slows compared with the water–binder ratio of the 0.22 test group.

The high modulus of elasticity of the PE fibers increases the ability of the ECCs to resist deformation and absorb energy. The fibers were dispersed at the cracked cross-section of the sample to inhibit the rapid development of its cracks. Although the low water–binder ratio resulted in uneven fiber dispersion and excessive bonding at the matrix/fiber interface, the high elastic modulus property could minimize the negative effect of the low water–binder ratio when many fibers were distributed across the cross-section.

**Figure 11:**
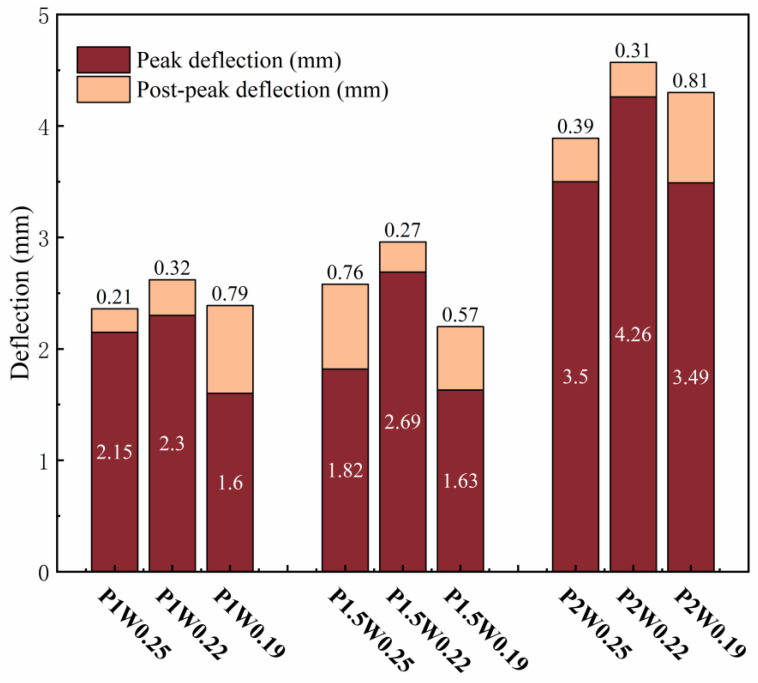
Deflection variation diagram.

The variation rule of deflection is shown in Figure 11, where peak deflection is the difference between the deflection value corresponding to the extreme point on the load–deflection curve, and postpeak deflection is the deflection value corresponding to the section of the descending curve from the peak deflection to 0.85 times the peak load. The peak deflection increases and then decreases with decreasing water–glue ratio. In the P2W0.22 test group, the maximum peak deflection reached was 4.26 mm; compared with 3.49 mm in the P2W0.19 test group, where the growth rate of peak deflection was 22.06%. The peak deflection shows a general growth trend with increasing fiber content. The maximum peak deflection is reached in the P2W0.22 test group, and its growth rate is 85.22% compared with that of the P1W0.22 test group, which indicates that an increase in the fiber content has a significant effect on the growth of the peak deflection of the ECCs.

When the water–binder ratio is reduced to 0.19, the cracked cross-section matrix and fibers are too tightly bonded, and the remainder of the matrix cannot produce new cracks. The damage continues to develop in the main crack, the stress at the fracture interface is borne by the fibers, the peak bending strength arrives earlier, and the peak deflection decreases. Moreover, the high modulus of elasticity of the PE fibers results in a water–binder ratio of 0.19 in the test group, which reaches the peak load, delays the expansion of the main crack, and continues to produce new small cracks, and the deflection continues to increase. All of the postpeak deflections at a water–binder ratio of 0.19 were larger than those at a water–binder ratio of 0.22, so it is necessary to pay attention to the whole process of bending changes in ECCs with low water–binder ratios.

#### 3.2.2. Bending Toughness

This test for the ECC bending toughness research was used in the Japan JSCE SF-4 [21] toughness analysis, the United States *ASTM C1609-12* [20] toughness analysis, and Zhang’s proposal for the evaluation of ECC bending toughness standards. Three methods were used to evaluate the bending toughness from different perspectives of this test and to compare their respective advantages and shortcomings.

(1) Japan *JSCE SF-4* Toughness Analysis

The *Japanese JSCE SF-4* [21] test standard uses a simple and applicable toughness factor (equivalent bending strength) to characterize the bending toughness of FRC with the following expression:

(7) $$f_{e}=\frac{\Omega_{L/150}L}{\delta_{L/150}bh^{2}}$$

where *Ω_L/150_* is the closed area with a mid-span deflection of *δ_L/150_*, (*δ_L/150_ = L/150*) and *f_e_* is the equivalent bending strength.

The advantage of the toughness evaluation method is that this evaluation method is not affected by the selection of the initial cracking point, and taking the solid k value to a fixed deflection does not affect the equivalent bending strength value in the strain hardening curve section. However, its shortcomings are also obvious: (1) the selection of deflection (*δ_L/150_ = L/150*) is more arbitrary, and there is no theoretical basis; (2) the fixed deflection to takes a small value; and (3) the toughness evaluation calculation method is related to the size of the sample. In addition, the deflection of the ECC sample is much greater than *L/150* (the test was set up to destroy the deflection of 1 mm).

(2) ASTM C1609-12 Toughness Analysis

The *ASTM C1609* [20] method is proposed based on the *ASTM C1018* [31] method, which eliminates concerns about the determination of the first crack point. The method proposes an equivalent bending strength and equivalent bending toughness ratio to evaluate the bending toughness of fiber–cement matrix composites, which is expressed as follows:

(8) $$f_{L/600}=\frac{P_{L/600}L}{bd^{2}}$$

(9) $$f_{L/150}=\frac{P_{L/150}L}{bd^{2}}$$

(10) $$R_{e,L/150}=\frac{150\Omega_{L/150}}{f_{L/150}bd^{2}}\times 100\%$$

where *P_L/600_* is the load at a deflection of *L/600*, *f_L/600_* is the bending strength at a deflection of *L/600*, Ω*_L/150_* is the area under the load deflection diagram for a deflection of *L/150*, and *R_e,L/150_* is the equivalent bending toughness ratio for a deflection of *L/150.*

This method is improved based on the *ASTM C1018* method, which eliminates the value of the first crack point and overcomes the *JSCE SF-4* method for the initial peak load. The peak load difference is reflected, but its shortcomings are also obvious: ① the final deflection is the relative fixed deflection; ② the calculation method is related to the size of the sample; and ③ the fixed deflection is taken as a small value. In addition, the *ASTM C1609* toughness evaluation method is the same as the *JSCE SF-4* method, and the small, fixed deflection makes it difficult to reflect the high deflection and toughness exhibited by the ECCs.

(3) Analysis of ECCs toughness evaluation methods

Referring to the above toughness indices and considering the flexural hardening behavior of ECCs, Zhang [27] proposed evaluating the bending toughness of ECC materials via the equivalent initial bending strength, initial energy factor, and postpeak bending toughness index. The expression is as follows:

(11) $$f_{e,m}=\frac{\Omega_{m}L}{\delta_{m}bh^{2}}$$

(12) $$E_{m}=f_{e,m}\times \frac{\delta_{m}}{L}=\frac{\Omega_{m}}{bh^{2}}$$

(13) $$I_{post}=\frac{\Omega_{post}}{\Omega_{m}+\Omega_{post}}$$

where *Ω_m_* is the zero to peak load area under the load–deflection diagram, *Ω_post_* is the area under the postpeak load–deflection map, *f_e,m_* is the equivalent initial bending strength, *E_m_* is the initial energy factor, and *I_post_* is the postpeak bending toughness index.

Zhang’s proposed method for calculating the bending toughness of an ECCs involves the use of an initial energy factor, which refers to the energy dissipation capacity per unit volume of the material, which can directly reflect the toughness of the ECCs. The authors also propose a postpeak bending toughness index for evaluating the residual toughness of the ECC. 

**Figure 12:**
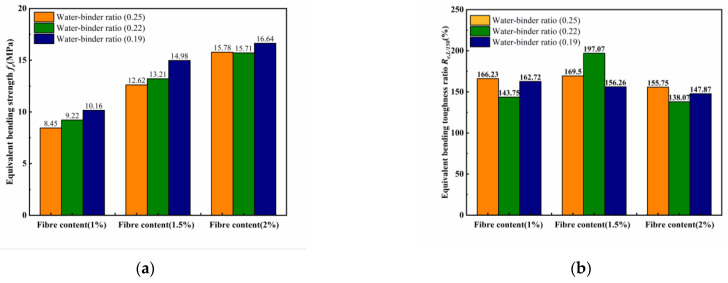

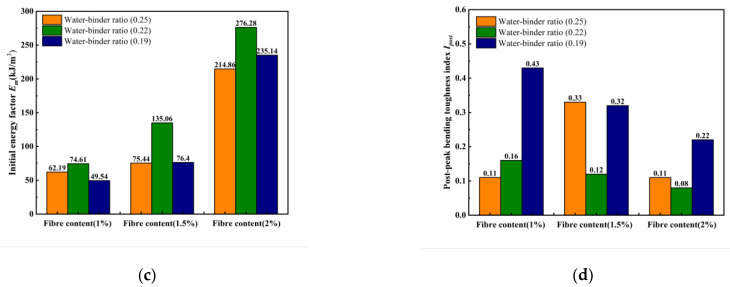
Evaluation results of different bending toughness values. (**a**) JSCE SF-4 Equivalent bending strength *fe.* (**b**) ASTM C1609-12 Equivalent bending toughness ratio *R_e,L/150_*. (**c**) Zhang’s Initial energy factor *E_m_*. (**d**) Zhang’s Postpeak bending toughness index *I_post_*.

The toughness evaluation method proposes a new index for evaluating the bending toughness of ECCs, which avoids the determination of the initial cracking point, has no effect on the sample size and proposes an evaluation method for the residual toughness. However, the shortcomings of this method are that it does not combine the prepeak toughness evaluation indices and postpeak toughness evaluation indices, and cannot comprehensively reflect the changes in the ECC bending toughness. The *JSCE—SF4* method (*f_e_*), ASTM C1609 method (*f_L/150_* and *R_e,L/150_*), and Zhang’s method were used to calculate the change rule of the bending toughness index, as shown in Figure 12.

Figure 12 shows that the *JSCE-SF4* method and the *ASTM C1609* method do not directly reflect the toughness of the ECCs, and that the bending toughness depends on the development rate of both the bending strength and the development rate of the sample deflection. For example, in Figure 12a for P2W0.19, the equivalent bending strength *f_e_* is maximal, and in Figure 12b for P1.5W0.22, the equivalent bending toughness ratio *R_e,L/150_* is maximal. Both values of the sample damage deflection are 2 mm, which is far from reaching the peak deflection of the ECCs sample. Due to the change in the water–binder ratio and the amount of fiber content, the area of the load–deflection map size is different, so the above two methods cannot fully reflect all the toughness of the ECCs. The initial energy factor *E_ms_* and postpeak bending toughness *I_post_* proposed by Zhang’s method can reflect the changes in the ECCs toughness more comprehensively. However, the changes in the water–binder ratio and fiber content make the peak deflection of the samples different. The corresponding postpeak deflection develops when the water–binder ratio is 0.19, which corresponds to the difference in postpeak energy dissipation. For example, the *E_m_* of P1W0.19 in Figure 12c reaches the minimum value of only 49.54 kJ/m^3^, but *I_post_* in Figure 12d reaches the maximum value of 0.43, which means that the area *Ω_post_* under the postpeak load–deflection diagram is 43% of the sum of the peak and postpeak areas, and the area Ω*_m_* under the peak load–deflection diagram is 57% of the sum of the peak and postpeak areas. However, the calculation of the initial energy factor *E_m_* only considers *Ω_m_* without calculating *Ω_post_,* then the results of the flexural toughness calculation will produce a great deviation, and it does not fully evaluate the overall toughness of ECCs.

(4) Energy factor toughness evaluation methods

Based on the above bending toughness evaluation method analysis, different water–binder ratios and fiber contents influence the prepeak and postpeak toughness differences in a sample. Thus, in this experimental analysis of the prepeak and postpeak energy dissipation unified calculations, the test results of the bending toughness index calculation peak load decreased to 0.85 times the corresponding deflection at the damage point of the sample were used to obtain energy factor Ems to analyze the change in the bending test. The calculation formula is as follows, and the results are shown in Figure 14.

(14) $$E_{ms}=\frac{\Omega_{ms}}{bh^{2}}$$

where *Ω_ms_* is the area at the deflection, corresponding to 0 to 0.85 times the peak load under the load–deflection diagram and also is the energy factor.

**Figure 13:**
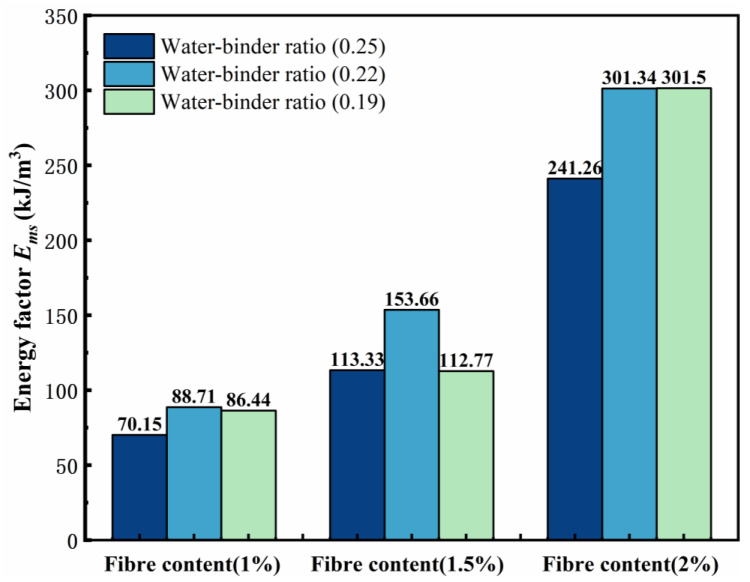
Plot of energy factor changes.

Figure 13 shows the variation rule of the energy factor. With a decreasing water–binder ratio, the ECCs of the different fiber contents at P1W0.22, P1.5W0.22, and P2W0.19 reached a maximum, with values of 88.71 kJ/m^3^, 153.66 kJ/m^3^, and 301.5 kJ/m^3^, respectively, and the water–binder ratio of 0.25 and reached a minimum, with values of 70.15 kJ/m^3^, 113.33 kJ/m^3^, and 241.26 kJ/m^3^, respectively. The energy factor of the samples at the same water–binder ratio tended to increase with increasing fiber content and reached a maximum at P2W0.19.

When the fiber content is unchanged, the pattern of the energy factor *E_ms_* is approximately the same. When the fiber content is 1%, with a decrease in the water–binder ratio, the Ems tends to increase and then decrease. When the fiber content is 1.5%, with a decrease in the water–binder ratio, the *E_ms_* also tends to increase and then decrease. When the fiber content is 2%, with a decrease in the water–binder ratio, the Ems continues to increase and reaches a maximum value of 301.50 kJ/m^3^ at P2W0.19, and the Ems of P2W0.22 is only 0.16 kJ/m^3^ lower than that of P2W0.19.

Notably, the initial energy factors Em of P2W0.22 and P2W0.19 are 276.28 kJ/m^3^ and 235.14 kJ/m^3^, respectively, with a large difference between the two, which is attributed to the excessive fiber–matrix cross-section bonding at P2W0.19, and the further development of the damage along the internal deleterious pores in the sample during the bending process, whereas P2W0.19 is further damaged by the fiber after the peak loading by the fiber. More energy is absorbed by the bridging effect, and the high elastic modulus of the PE fibers plays a large role, and *I_post_* continues to improve, which is consistent with the pattern exhibited by the remaining two fiber contents in Figure 13. At the same water–binder ratio, the energy factor *E_ms_* of the ECCs increases significantly with increasing fiber content, and the fibers absorb most of the damage energy in the bending deformation of the sample.

### 3.3. Response Surface Methodology Analysis

The test mix ratios obtained from the previous tensile and bending tests are optimal for single properties, but they lack universality and economic efficiency in application. Therefore, using Design-Expert 12 software, based on the experimental results, taking tensile strength, tensile toughness, bending strength, and bending toughness as response values, the effects of different water–binder ratios and fiber contents on the tensile strength, tensile toughness, bending strength, and bending toughness of ECCs are studied. Through multi-objective optimization, the optimal water–binder ratios and fiber contents under different conditions are found, achieving the goal of combining high strength and high toughness of ECCs. The three-factor and three-level experimental design was carried out with two factors, with the water–binder ratio and fiber content as independent variables. The experimental factors and levels are shown in Table 8, and the experimental design scheme and results are displayed in Table 9.

#### 3.3.1. Tensile Strength Response Surface Modeling

The tensile strength data were fitted to a binomial polynomial, using the water–binder ratio and PE fiber admixture as response variables and the tensile strength as the response value. The resulting fitting equations for tensile strength are as follows:

(15) $$F_{c}=-51.65494+509.56287A+4.41298Β+23.50AB-1245.02924A^{2}-3.02211{Β}^{2}.$$

Table 10 displays the ANOVA test results for the tensile strength model. The F-value and *p* value jointly determine the significance of this regression model, meaning that a larger F value corresponds to a smaller *p* value. As shown in Table 10, the tensile strength model has a *p* value of less than 0.05, with significant results, whereas its misfit model displays a *p* value greater than 0.05, with an insignificant effect. This finding indicates the good fit and reliability of the model, paving the way for the subsequent analysis step.

**Figure 14:**
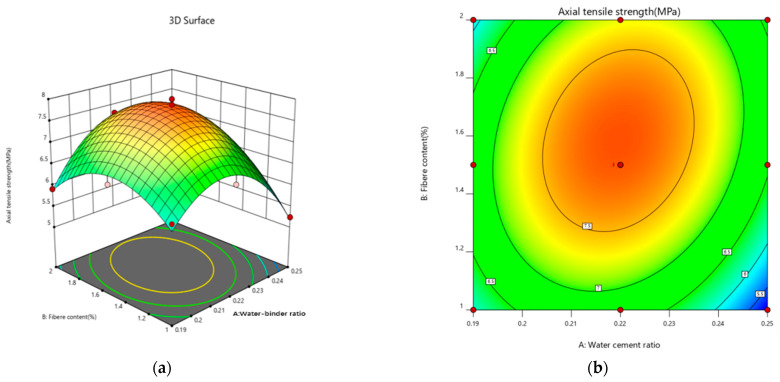
Interaction effect curves of different factors on the tensile strength. (**a**) Tensile Strength 3D Response Plot. (**b**) Tensile strength contour map. In the contour plot of the response surface method, blue areas represent lower yields, green areas represent medium yields, and red areas represent higher yields.

Figure 14 shows the 3D response surface and contours of the effects of the water–binder ratio and fiber content on the tensile strength. There is a distinct trend in the tensile strength response surface. The water–binder ratio and fiber content play a large role in the response surface. The tensile strength is lowest when the water–binder ratio is 0.25 and the fiber content is 1%. Reducing the water–binder ratio densifies the internal structure of the sample and strengthens the fiber–matrix cross-section bond, which becomes an important factor in tensile strength enhancement. As the water–binder ratio decreases and the fiber content increases, the tensile strength of a sample initially increases before it decreases. However, excessive fiber content leads to a decrease in the degree of hydride bonding between the fiber and matrix cross-sections, an excessively large fiber–matrix cross-section bond, and internal pore space damage, which accelerates the damage process and reduces the tensile strength of the sample.

#### 3.3.2. Tensile Toughness Response Surface Modeling

The tensile toughness data were entered into Design-Expert 12 software for analysis, and the tensile toughness data were fitted with binomial polynomials, using the water–binder ratio and PE fiber admixture as response variables and tensile toughness as the response value. The resulting fitting equations for tensile toughness are as follows:

(16) $$F_{c}=-201.15263+900.47661A+152.73579Β-21.0AΒ-1962.57310A^{2}-41.14526{Β}^{2}.$$

Table 11 displays the ANOVA results, which tested the significance of the tensile toughness model. The F value and *p* value jointly determine the significance of this regression model, meaning that a larger F value corresponds to a smaller *p* value. If the *p* value of the tensile toughness model in Table 11 is less than 0.05 and the result is significant, and if the *p* value of its misfit model is greater than 0.05 and the effect is not significant, this indicates that the model is well fitted and reliable, allowing for the next step of the analysis.

**Figure 15:**
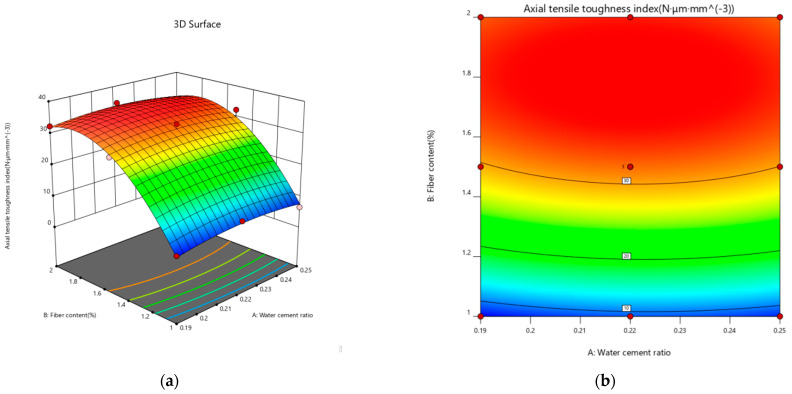
Interaction effect curves of different factors on the tensile toughness. (**a**) Tensile Toughness 3D Response Surface Map. (**b**) Tensile toughness contour map.

Figure 15 shows how the water–binder ratio and fiber content affect the tensile toughness. Interestingly, the fiber content has a large effect on the tensile toughness, whereas the water–binder ratio has a small effect on the tensile toughness, and the same goes for the change in toughness in the tensile test, in which the fiber content has a significant effect on the growth of tensile toughness of ECC. With the increase in fiber content, the overall tensile toughness of the specimen is an increasing trend. In 2.0% fiber content, the tensile toughness values have reached the maximum value. The deformation ability of a sample is affected by the damage to the cross-section of the connection bearing, and the fiber–matrix cross-section of the bond increases with decreasing water–binder ratio. As the water–binder ratio decreases, the damage to the cross-sections of the fiber shifts from half pull-outs to full pull-outs, causing the tensile toughness to initially increase and then decrease. As the fiber content increases, more fibers damage the cross-section of the sample, increasing its deformation capacity. As the fibers continue to increase, the bonds in the tensile interval shrink, leading to an uneven distribution of fibers and fiber entanglement, which accelerates the destruction of the sample and decreases its tensile toughness.

#### 3.3.3. Bending Strength Response Surface Modeling

The bending strength data were input into Design-Expert 12 software for analysis, and the bending strength data were fitted with binomial polynomials, using the water–binder ratio and PE fiber admixture as response variables and the bending strength as the response value. The fitting equations for the bending strength are as follows:

(17) $$F_{c}=-34.99012+306.54094A+26.68070Β-17.0AΒ-776.60819A^{2}-3.63579{Β}^{2}.$$

Table 12 displays the ANOVA test results for the bending strength model. The F value and *p* value jointly determine the significance of this regression model, meaning that a larger F value corresponds to a smaller *p* value. The bending strength model in Table 12 has a *p* value of less than 0.05 with significant results, and its misfit model has a *p* value of more than 0.05 with an insignificant effect. This finding shows that the model is well fitted and reliable, from which the next step in the analysis is carried out.

**Figure 16:**
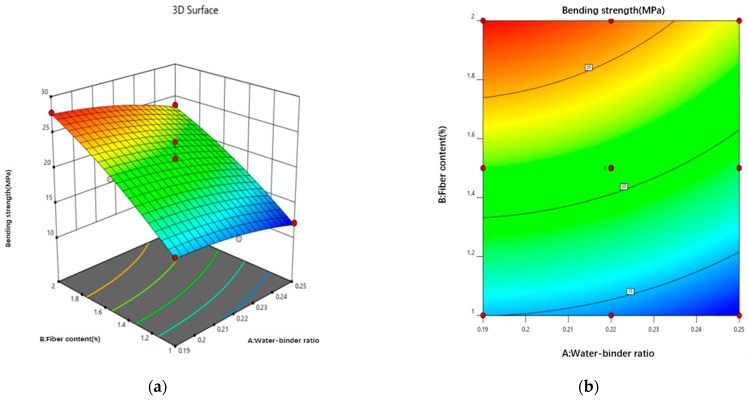
Interaction effect curves of different factors on the bending strength. (**a**) 3D response surface plot of the bending strength. (**b**) Bending strength contour map.

Figure 16 provides much information about how the water–binder ratio and fiber content points affect the bending strength. When the water–binder ratio is 0.19 at the lowest design factor and 2% at the highest design factor, the bending strength peaks. With increasing fiber content, the bending strength of the sample significantly increased, because in the bending process, the lower part of the sample cracked, and then gradually cracked from the lower end through to the entire damage section. In addition, when the fibers at both ends bridge, the fiber content continues to increase so that the fibers fill in the crack damage section, slowing down the development of cracks. More fibers participate in the damage process, resulting in multiseam cracking, and the bending strength continues to improve. When the W/B ratio is as low as 0.19, the highest design factor is 2%. As the water–binder ratio increases, the bending strength gradually decreases, because the sample is experiencing compression in the upper part of the bending process, a tensile state in the lower part, and a slow upward shift in the central axis during the destruction process. Once the lower part of the tensile damage develops, the fiber–matrix cross-section primarily plays the role of the bond, mirroring the tensile destruction mechanism of the tensile test.

#### 3.3.4. Bending Toughness Response Surface Modeling

The energy factor Ems data were entered into the Design-Expert 12 software for analysis, and the energy factor Ems was fitted to a binomial polynomial, using the water–binder ratio and PE fiber admixture as response variables and the energy factor Ems as the response value. The resulting fitting equation for the energy factor Ems is as follows:

(18) $$F_{c}=-1423.02746+15173.66520A-267.11526Β-732.50AΒ-32947.66082A^{2}+209.28842{Β}^{2}$$

Table 13 displays the ANOVA test results for the energy factor E_ms_ model. The F value and *p* value jointly determine the significance of this regression model, meaning that a larger F value corresponds to a smaller *p* value. The energy factor Ems model in Table 13 has a *p* value of less than 0.05 with significant results, and its misfit model has a *p* value of more than 0.05 with insignificant effects, which indicates that the model is well-fitted and reliable, from which the next step of the analysis is carried out.

**Figure 17:**
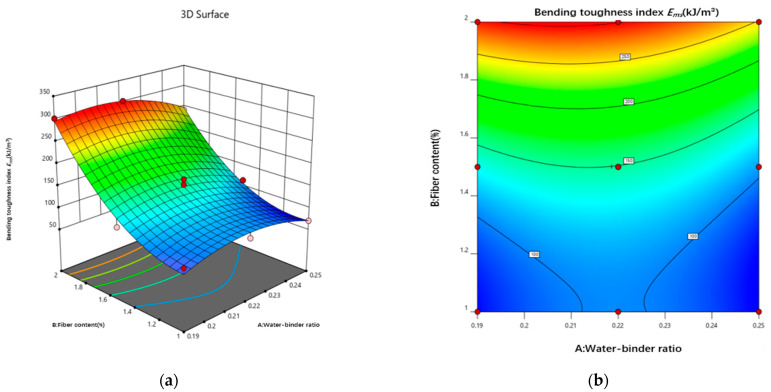
Interaction effect curves of different factors on the bending toughness. (**a**) 3D response surface plot of energy factor Ems. (**b**) Contour map of energy factor Ems.

Figure 17 shows the 3D response surface and contour plot of the effects of the water–binder ratio and fiber content on the energy factor. The energy factor E_ms_ is significantly influenced by the fiber content; it increases with a decreasing water–binder ratio, decreases and increases with an increasing fiber content, and rapidly increases above a 1.4% fiber content. Additionally, an increase in fiber slows down the development of cracks in the damage section, causing them to develop into multiseam cracks. The increase in fibers slows down the development of cracks in the damage section, causing them to develop into multiseam cracks, thereby increasing the damage deflection of the sample and increasing its energy absorption capacity. The decrease in the water–binder ratio strengthens the bond of the fiber–substrate cross-section, reaching the maximum value of the energy factor E_ms_ in the interval 0.21–0.23. This finding indicates that the bond of the fiber–substrate cross-section is the largest in this interval, maximizing the high elastic modulus of the fibers to aid in energy absorption by the sample.

#### 3.3.5. Multi-Objective Optimization Scheme

Design-Expert 12 software was used to optimize and predict the experimental data to determine the optimal process matching scheme for the ECC.

Table 14 displays the optimization results. This occurs by taking the maximum values of tensile strength, bending strength, tensile toughness, and the energy factor *E_ms_* as the objectives. When the water–binder ratio is 0.22 and fiber content is 1.9%, the ideal values for tensile strength, bending strength, tensile toughness, and energy factor E_ms_ are 7.336 MPa, 25.93 MPa, 34.02 kJ/m^3^, and 289.84 kJ/m^3^, respectively. A verification test was carried out with this mix ratio. The tensile strength, flexural strength, tensile toughness, and energy factor *E_ms_* of the measured group were 7.53 MPa, 25.05 MPa, 35.12 kJ/m^3^, and 293.36 kJ/m^3^ respectively. Compared with the predicted results, the tensile strength increased by 2.64%, the bending strength decreased by 3.39%, the tensile toughness increased by 3.22%, and the energy factor E_ms_ increased by 1.21%. The bending strength was slightly lower than the predicted value in the measured group, and the tensile strength, tensile toughness, and energy factor *E_ms_* were all improved to varying degrees compared to the predicted resultant values.

The mix ratio optimized based on the response surface method means that when the water–binder ratio is 0.22 and the fiber content is 1.9%, the experimental group with the optimal comprehensive performance of tensile strength, bending strength, tensile toughness, and bending toughness *E_ms_* can be obtained. This basically achieves the goal of combining high strength and high toughness of ECCs.

## 4. Conclusions

In the study, ECCs, the water–binder ratio, and the fiber content were used as the research objects.

(1) The water–binder ratio and fiber content affect growth of the ECC tensile strength in the P1.5W0.22 test group, which reaches a maximum tensile strength of 7.87 MPa. The impact of the fiber content on the ECC tensile toughness and elongation was significant, with a 2% fiber content reaching a maximum. The P2W0.22 test group reached the maximum tensile toughness of 34.50 kJ/m^3^. The P2W0.25 test group reached the maximum elongation of 6.52%.
(2) Through the scanning electron microscope test, it can be observed that with the decrease in the fiber content ratio, the destructive cross-section of the fiber presents the majority of the pull-out, which progresses as follows: half of the pull-out; half of the pull-off; a few pull-out; then most of the pull-off. This is in line with the change in the rule of the tensile strength, which suggests that with the reduction in the fiber content ratio, the fiber/matrix cross-section adhesion presents an initial rise and then fall, according to the rule of law. This occurs in the fiber content ratio of 0.22 to reach the maximum.
(3) By comparing three bending toughness evaluation indicators, it was found that the JSCE SF-4 test standard and the ASTM C1609 standard are not fully adequate for calculating the bending toughness of high-deflection ECC, and cannot completely reflect the high toughness of ECC. Zhang’s toughness evaluation method analyzed and evaluated the prepeak and postpeak toughness indicators, but due to the reduction in the water–binder ratio causing the specimen to reach the peak load earlier, and the inability to jointly analyze the prepeak and postpeak phases, it cannot fully reflect the overall toughness of the ECC specimen failure. Therefore, this experiment proposes the energy factor Ems to eliminate the evaluation of prepeak and postpeak bending toughness and uses the deflection area at 0 to 0.85 times the peak load to calculate the energy factor Ems, thereby evaluating the bending toughness of ECC. The energy factor Ems of the P2W0.19 test group reached a maximum value of 301.5 kJ/m^3^, with a growth rate of 248.80% in bending toughness compared to the P1W0.19 test group.
(4) The fiber content has a significant effect on the growth of ECC bending strength and peak deflection, both of which show excellent performance at 2% fiber content. Among them, the P2W0.19 test group reached the maximum bending strength of 27.79 MPa, and the P2W0.22 test group reached the maximum peak deflection of 4.26 mm. The growth rates of bending strength and peak deflection were 82.59% and 85.22%, respectively, compared with those of the same water–binder ratio and 1% fiber content.
(5) Under the combined influence of two crucial factors, namely the water–binder ratio and fiber content, the response surface methodology was employed to meticulously analyze their impacts on several key mechanical properties, including tensile strength, bending strength, tensile toughness, and bending toughness Ems. By setting the maximum values of tensile strength, bending strength, tensile toughness, and bending toughness *E_ms_* as the optimization targets, the optimal predicted values of 7.34 MPa, 25.9 MPa, 34.02 kJ/m^3^, and 289.84 kJ/m^3^ were achieved when the water–binder ratio was precisely adjusted to 0.22 and the fiber content was maintained at 1.9%. Subsequently, a verification experiment was conducted using this specific mix ratio. The measured values of the aforementioned mechanical properties were obtained as 7.53 MPa, 25.05 MPa, 35.12 kJ/m^3^, and 293.36 kJ/m^3^, respectively. Compared with the predicted values, these measured values exhibited improvements of 2.64%, −3.39%, 3.22%, and 1.21%. Overall, the optimized mix ratio design based on the response surface method has essentially realized the desirable combination of high strength and high toughness in ECCs materials.

## Figures and Tables

**Table 1:** Cumulative range of material particle sizes.

Particle Size (μm)	0.46	1.13	2.75	5.21	9.86	14.5	21.1	31.3	51.8	98.1	250
Cement (%)	0	3.5	12.2	25	41.5	53.7	68.2	82.9	96.2	100	100
Fly ash (%)	0	2.3	18.5	36.2	52.4	62.8	72.5	80.9	97.1	100	100
Silica fume (%)	1.7	16.5	43.7	75.6	96.1	100	100	100	100	100	100
quartz sand (%)	0	0	0	0	0	0	0	0	0	26	100

**Table 2:** PE fiber performance indices.

Items	Density	Diameter	Lengths	Modulus of Elasticity
	(g·cm^3^)	(mm)	(mm)	(GPa)
Test results	0.97	0.025	18	116

**Table 3:** Cement performance indices.

Items	Density	Specific Surface Area	Condensation Time (min)		Bending Strength (MPa)	
	(kg/m^3^)	(m^2^/kg)	Initial Condensation	Final Condensation	7 d	28 d
Test results	3155	3600	200	300	4.7	9.2

**Table 4:** Fly ash performance indices.

Items	Density	Firing Vector (physics)	Moisture Content	Fineness	Packing Density
	(g/cm^3^)	(%)	(%)	(%)	(g/cm^3^)
Test results	2.55	2.8	0.85	16	1.12

**Table 5:** Silica fume performance indices.

Items	Chloride Ion Content	Scorch Reduction	Water-Demand Ratio	28 d Activity Indice	SiO_2_
	(%)	(%)	(%)	(%)	(%)
Test results	0.01	1.48	112	105	1.12

**Table 6:** Quartz sand performance indices.

Items	Packing Density	Breakage Rate	Wear Rate	Porosity	Proportion	Mohs’ Hardness
	(g/cm^3^)	(%)	(%)	(%)	(g/cm^3^)	
Test results	1.65	0.51	0.35	43	2.66	7.5

**Table 7:** ECC mixing ratio.

Sample Number	Cement (kg/m^3^)	Fly Ash (kg/m^3^)	Silica Fume (kg/m^3^)	Quartz Sand (kg/m^3^)	PE (%)	Water-Binder Ratio	Water (kg/m^3^)
P1W0.25	824	357	51	703	1	0.25	308
P1W0.22						0.22	271
P1W0.19						0.19	234
P1.5W0.25	824	357	51	703	1.5	0.25	308
P1.5W0.22						0.22	271
P1.5W0.19						0.19	234
P2W0.25	824	357	51	703	2	0.25	308
P2W0.22						0.22	271
P2W0.19						0.19	234

**Table 8:** Selection of factors and levels for response surface analysis.

Factor	Level		
	−1	0	1
A: water–binder ratio	0.19	0.22	0.25
B: Fiber content/%	1	1.5	2

**Table 9:** Experimental design scheme and results.

Sample Number	Factor		Tensile Strength	Bending Strength	Tensile Toughness	*E_ms_*
	A	B	(MPa)	(MPa)	(N·μm·mm^−3^)	(kJ/m^3^)
1	0.19	1%	6.23	15.22	7.15	86.44
2	0.19	1.5%	6.54	21.81	26.83	112.77
3	0.19	2%	5.92	27.79	34.34	301.50
4	0.22	1%	6.54	13.73	9.58	86.44
5	0.22	1.5%	7.87	21.40	31.15	153.66
6	0.22	2%	7.30	25.71	34.50	301.34
7	0.25	1%	5.24	12.15	6.54	70.15
8	0.25	1.5%	6.57	18.05	32.29	113.33
9	0.25	2%	6.34	23.70	30.47	241.26

**Table 10:** Analysis of variance for the tensile strength.

Sample Number	Square Sum	Degrees of Freedom	Squared Difference	F-Value	*p*-Value	Significance
mold	7.16	5	1.43	34	0.0007	significant
A-A	0.0486	1	0.0486	1.15	0.3318	
B-B	0.4004	1	0.4004	9.51	0.0274	
AB	0.497	1	0.497	11.8	0.0185	
A^2^	3.18	1	3.18	75.53	0.0003	
B^2^	1.45	1	1.45	34.34	0.0021	
residual	0.2106	5	0.0421	-	-	
lost item	0.1273	3	0.0424	1.02	0.5299	insignificant
absolute error	0.0833	2	0.0416	-	-	
total deviation	7.37	10	-	-	-	

**Table 11:** Tensile toughness ANOVA.

Sample Number	Square Sum	Degrees of Freedom	Squared Difference	F-Value	*p*-Value	Significance
mold	1237.71	5	247.54	64.68	0.0002	significant
A-A	0.1601	1	0.1601	0.0418	0.846	
B-B	913.65	1	913.65	238.73	<0.0001	
AB	0.3969	1	0.3969	0.1037	0.7605	
A^2^	7.9	1	7.9	2.07	0.2102	
B^2^	268.05	1	268.05	70.04	0.0004	
residual	19.14	5	3.83	-	-	
lost item	11.12	3	3.71	0.925	0.557	insignificant
absolute error	8.02	2	4.01	-	-	
total deviation	1256.85	10	-	-	-	

**Table 12:** Analysis of variance for the bending strength.

Sample Number	Square Sum	Degrees of Freedom	Squared Difference	F-Value	*p*-Value	Significance
mold	241.85	5	48.37	18.76	0.003	significant
A-A	19.87	1	19.87	7.71	0.0391	
B-B	217.2	1	217.2	84.26	0.0003	
AB	0.2601	1	0.2601	0.1009	0.7636	
A^2^	1.24	1	1.24	0.4801	0.5192	
B^2^	2.09	1	2.09	0.812	0.4088	
residual	12.89	5	2.58	-	-	
lost item	1.37	3	0.4561	0.0792	0.9654	insignificant
absolute error	11.52	2	5.76	-	-	
total deviation	254.73	10	-	-	-	

**Table 13:** Energy factor Ems ANOVA.

Sample Number	Square Sum	Degrees of Freedom	Squared Difference	F-Value	*p*-Value	Significance
mold	68,812.58	5	13,762.52	56.39	0.0002	significant
A-A	961.91	1	961.91	3.94	0.1039	
B-B	59,760.24	1	59,760.24	244.86	<0.0001	
AB	482.9	1	482.9	1.98	0.2185	
A^2^	2227.55	1	2227.55	9.13	0.0294	
B^2^	6935.26	1	6935.26	28.42	0.0031	
residual	1220.28	5	244.06	-	-	
lost item	881.99	3	294	1.74	0.3855	insignificant
absolute error	338.29	2	169.15	-	-	
total deviation	70,032.86	10	-	-	-	

**Table 14:** Response Surface Prediction and Measured Results.

PE (%)	Water– Cement Ratio	Tensile Strength (MPa)		Bending Strength (MPa)		Tensile Toughness g(kJ/m^3^)		Energy Factor *E_ms_* (kJ/m^3^)	
1.9	0.22	predicted value	measured value	predicted value	measured value	predicted value	measured value	predicted value	measured value
		7.34	7.53	25.93	25.05	34.02	35.12	289.84	293.36

## Data Availability

The data used in the article can be obtained from the author here.
